# Expanding the repertoire of secretory peptides controlling root development with comparative genome analysis and functional assays

**DOI:** 10.1093/jxb/erv346

**Published:** 2015-07-20

**Authors:** Sarieh Ghorbani, Yao-Cheng Lin, Boris Parizot, Ana Fernandez, Maria Fransiska Njo, Yves Van de Peer, Tom Beeckman, Pierre Hilson

**Affiliations:** ^1^Department of Plant Systems Biology, VIB, 9052 Ghent, Belgium; ^2^Department of Plant Biotechnology and Bioinformatics, Ghent University, 9052 Ghent, Belgium; ^3^Genomics Research Institute, University of Pretoria, Hatfield Campus, Pretoria 0028, South Africa; ^4^Institut Jean-Pierre Bourgin, INRA, AgroParisTech, CNRS, Université Paris-Saclay, Saclay Plant Sciences, INRA, route de Saint-Cyr, 78026 Versailles, France

**Keywords:** Crops, genome annotation, meta-analysis, plant development, signalling peptide.

## Abstract

Plant genomes encode numerous small secretory peptides (SSPs) whose functions have yet to be explored. Based on structural features that characterize SSP families known to take part in postembryonic development, this comparative genome analysis resulted in the identification of genes coding for oligopeptides potentially involved in cell-to-cell communication. Because genome annotation based on short sequence homology is difficult, the criteria for the *de novo* identification and aggregation of conserved SSP sequences were first benchmarked across five reference plant species. The resulting gene families were then extended to 32 genome sequences, including major crops. The global phylogenetic pattern common to the functionally characterized SSP families suggests that their apparition and expansion coincide with that of the land plants. The SSP families can be searched online for members, sequences and consensus (http://bioinformatics.psb.ugent.be/webtools/PlantSSP/). Looking for putative regulators of root development, *Arabidopsis thaliana* SSP genes were further selected through transcriptome meta-analysis based on their expression at specific stages and in specific cell types in the course of the lateral root formation. As an additional indication that formerly uncharacterized SSPs may control development, this study showed that root growth and branching were altered by the application of synthetic peptides matching conserved SSP motifs, sometimes in very specific ways. The strategy used in the study, combining comparative genomics, transcriptome meta-analysis and peptide functional assays *in planta*, pinpoints factors potentially involved in non-cell-autonomous regulatory mechanisms. A similar approach can be implemented in different species for the study of a wide range of developmental programmes.

## Highlight

Comparative genome analysis combined with transcriptome mining identified secreted peptides possibly involved in root development. When applied on *Arabidopsis* roots, selected candidates affected growth or branching in specific ways.

## Introduction

Plants are complex organisms that consist of distinct cell types organized in tissues. Separate plant organs as well as neighbouring cells exchange a wide range of signals to coordinate development and respond to environmental stimuli. However, the phytohormones that had initially been recognized to control plant growth are relatively few in number. In recent years, peptides secreted into the apoplast by plant cells have also been identified as extracellular signals involved in various biological processes, including development (Grienenberger and Fletcher, 2015; Murphy *et al.*, 2012). These bioactive molecules are referred to hereafter as small secretory peptides (SSPs). Most SSPs are synthesized as preproproteins from which the signal sequence is cleaved upon targeting in the endoplasmic reticulum and further processed by successive proteolytic cleavages through the secretory pathway. Subclasses of cysteine-poor SSPs also undergo additional post-translational modifications, among which proline hydroxylation, hydroxyproline arabinosylation, and tyrosine sulfation have been documented (Matsubayashi, 2014).

Because plants are sessile organisms, they have evolved a remarkable developmental plasticity in order to adapt to a wide range of ecological niches (Guyomarc’h *et al.*, 2010). For example, embryonic roots grow and branch to produce the entire root system through a finely coordinated developmental process that integrates endogenous and environmental cues. Multiple reports have shown that SSPs play an important role in meristem establishment and maintenance, cell division, lateral root (LR) initiation, development, and emergence (recently reviewed in Delay *et al.*, 2013*a*; Somssich and Simon, 2012).

In *Arabidopsis*, the LR primordium (LRP) is formed through successive coordinated cell division events, initiated with the first asymmetric division of the pericycle founder cells, and leading to the emergence of the LR (Malamy and Benfey, 1997). The study of promoter–reporter constructs revealed that *GOLVEN* (*GLV*) genes are expressed differentially in specific cells and at specific stages during this developmental programme (Fernandez *et al.*, 2013). Overproduction of GLV peptides resulted in a decreased number of LRs and perturbed cell divisions in LRP (Fernandez *et al.*, 2013; Meng *et al.*, 2012). Besides their known role in floral organ abscission, the INFLORESCENCE DEFICIENT IN ABCISSION (IDA) peptide, together with its receptors HAESA (HAE) and HAESA-Like 2 (HSL2), have recently been shown to be involved in LR emergence (Kumpf *et al.*, 2013). Moreover, a role in LR development has been proposed for the C-TERMINALLY ENCODED PEPTIDE 1 (CEP1) in *Arabidopsis* and *Medicago truncatula,* as demonstrated by the LR inhibition resulting from *CEP1* overexpression or the application of the peptide (Delay *et al.*, 2013*b*; Imin *et al.*, 2013; Ohyama *et al.*, 2008). Finally, a regulatory module has been identified in which the ERF115 transcription factor, specifically expressed in the root quiescent centre (QC), acts as a rate-limiting factor of cell division and is a direct activator of a phytosulfokine peptide (PSK5) known to control cell division (Heyman *et al.*, 2013).

Previous studies suggest that plant genomes contain more SSP genes than those that have been identified until now and whose function remains to be established (Hanada *et al.*, 2013; Lease and Walker, 2006, 2010; Okamoto *et al.*, 2014; Silverstein *et al.*, 2007). Indeed, the annotation of genes coding for SSPs is problematic because they harbour fewer characteristics of protein-coding sequences than larger genes and homology search linking sequence and function is restricted to domains coding for just a few amino acid residues conserved across SSP families. Therefore, bioinformatic pipelines relying simply on sequence homology do not accurately predict SSP genes (Oelkers *et al.*, 2008). Furthermore, hypothetical short open reading frames (ORFs) may arise by chance, albeit without function. Therefore, small ORFs are often under-predicted or systematically removed in genome annotation projects, as was the case in early releases of the *Arabidopsis* genome. Additionally, the detection of mature SSPs from crude plant tissue extracts is difficult because they are present at very low physiological concentrations (nanomolar range) and are generally masked by degradation products of larger and much more abundant proteins. Hence, it is likely that only a portion of the functional SSPs are known to date.

This study presents a refined method to identify unknown SSPs encoded in plant genomes without prior knowledge of their sequence. On the assumption that SSPs share short conserved oligopeptide stretches, the authors fine-tuned pattern recognition algorithms based on known plant SSP regulators and expanded SSP families to 32 species, including crops. The authors further investigated whether previously uncharacterized SSPs might be involved in root development and showed that some of the corresponding genes were expressed in specific cell types and at particular stages of LR initiation. Finally, the study demonstrated that synthetic peptides matching these SSP conserved motifs strongly alter LR emergence.

## Materials and methods

### Selection of short proteins with signal peptide

As the *de novo* detection of secretory peptides is sensitive to the quality of the gene models, five sequenced plant genomes with consistently improved annotations were selected: *Arabidopsis thaliana* (TAIR10), rice (*Oryza sativa*; IRGSPbuild5 and MSU6.1) (Ouyang *et al.*, 2007; Tanaka *et al.*, 2008); poplar (*Populus trichocarpa*; JGI v156) (Tuskan *et al.*, 2006); grapevine (*Vitis vinifera*; Genoscope v1) (Jaillon *et al.*, 2007) and maize (*Zea mays*; ZmB73_5a) (Schnable *et al.*, 2009). For all five species, genome annotations had been updated at least once after their initial release at the time this analysis was conducted, thus providing quality curated data. Two rice genome annotations were processed because their annotation of small predicted proteins was complementary. Only protein sequences of less than 200 amino acids in length were kept for further analysis. The authors searched for the presence of the signal peptide in the amino-terminal domain by using SignalP v3.0 software (Bendtsen *et al.*, 2004). The signal peptide was predicted with the neural network or hidden Markov model (HMM) profile.

### *De novo* conserved secretory motif detection

The last 50 amino acids from the candidate secretory peptides were searched against each other by using the FASTA program (Pearson, 2000) with the BLOSUM50 scoring matrix to detect mildly related sequences. Second, the all-against-all FASTA search results were subjected to the Markov Cluster Algorithm (MCL version 09-308, inflation value 1.5) (Enright *et al.*, 2002) to identify the sequences into clusters based on the e-value. Special attention was paid to the inflation point in the MCL algorithm because it controls the connectivity between related protein subgroups and the main challenge in the delineation of secretory peptide families is the weak sequence similarity between members. Third, sequences in each cluster were aligned by using the multiple alignment program MUSCLE (Edgar, 2004); non-aligned gaps and non-conserved positions in the multiple alignment were removed based on the BLOSUM62 scoring matrix. Fourth, based on the remaining conserved region, each cluster was represented by a HMM profile with hmmbuild and hmmcalibrate from the HMMER (v2.3.2) package (http://hmmer.wustl.edu/). Fifth, singleton sequences that did not cluster in the previous MCL clustering were searched (hmmersearch) against the HMM profiles to identify the most closely related clusters. When an additional sequence was identified in a cluster, this sequence was combined with the pre-existing ones in that cluster, and the procedure was reinitiated from step 3. We considered the search for a cluster to be completed once no sequence could be added to it.

The HMM profile of each cluster was compared against all HMM profiles by using the Profile Comparer (PRC) (Madera, 2008). Then, the higher-order relationship of the clusters was determined with the MCL algorithm based on the e-values calculated with PRC. To inspect the shared conserved motif of candidate secretory cluster pairs, ‘LogoMat-P’ (Schuster-Böckler and Bateman, 2005) was applied to generate the pairwise HMM logos. A group of clusters linked by the PRC program was considered to be one putative secretory family (http://bioinformatics.psb.ugent.be/webtools/PlantSSP/browse.php).

### Analysis of SSP sequences across plant genomes

The genome annotations of 32 photosynthetic organisms were downloaded from Phytozome or genome-specific databases (Supplementary Table 1, available at *JXB* online). These included updated versions of the reference species genomes selected for the initial clustering, most importantly a unified genome for rice (Kawahara *et al.*, 2013) and an updated genome assembly and annotation for poplar. Protein sequences were filtered with the same criteria as applied to the reference species genomes: protein sequence shorter than 200 amino acids with signal peptide in the N-terminal detected by SignalP. In total, 75,970 proteins were suitable for screening for the SSP signature, among which 35,875 contained SSP motifs as defined in the library created with the reference species (hmmpfam e-value 0.05) (http://bioinformatics.psb.ugent.be/webtools/PlantSSP/).

### Microarray data normalization and compendium analysis

Transcriptome datasets were retrieved as Gene Expression Omnibus accessions: GDS1515 (Vanneste *et al.*, 2005), GSE42896 (De Rybel *et al.*, 2012), GSE6349 (De Smet *et al.*, 2008), and GSE8934 (Brady *et al.*, 2007) for the phloem and the xylem pole pericycle expression files. The full pericycle expression data, based on the J2661 *Arabidopsis* marker line, were a kind gift (Levesque *et al.*, 2006). Array data were normalized with the robust multiarray average algorithm (Irizarry *et al.*, 2003) and the absolute values, fold change (FC), and pairwise *P*-values were determined with the affylmGUI R package (Smyth, 2004) without adjustment. Two-factor analysis of variance (ANOVA) *P*-values were computed with the MultiExperiment Viewer (http://www.tm4.org/mev.html). Affymetrix probe sets were assigned to AGI gene ID according to the affy_ATH1_array_elements-2010-12-20.txt file from TAIR (www.Arabidopsis.org). Ambiguously assigned genes (multiple gene identifiers for one probe set) and microarray controls were discarded. Genes were considered significantly regulated in specific experiments when the following criteria were fulfilled: absolute FC ≥ 1.5, *P* ≤ 0.01 for at least one of the pairwise comparisons (0–2, 2–6, 0–6h) upon LR induction in the control plants, and a two-factor ANOVA *P* ≤ 0.01 for the interaction between treatment and genotype (Vanneste *et al.*, 2005); absolute FC ≥ 1.5, *P* ≤ 0.01 for at least one of the pairwise comparisons (0–2, 2–6, 0–6h) for both compounds [1-naphthaleneacetic acid (NAA) and naxillin] during the time course upon the LR induction system (De Rybel *et al.*, 2012); absolute FC ≥ 1.5, *P* ≤ 0.01 for at least one of the pairwise comparisons (0–2, 2–6, 0–6h) during the time course upon LR initiation in the sorted pericycle cells (De Smet *et al.*, 2008); absolute FC ≥ 1.5, *P* ≤ 0.01 for at least one of the pairwise comparisons (xylem pole pericycle vs. phloem pole pericycle, xylem pole pericycle vs. full pericycle, full pericycle vs. phloem pole pericycle) and similar positive or negative sign for all the pairwise comparisons (Parizot *et al.*, 2012). Additionally, a radial layer specificity was determined as described by Brady *et al.* (2007) and a gene was tagged when specifically expressed in the xylem or phloem pericycle pole, or in the primordium. Furthermore, an oscillation cluster association was determined as described by Moreno-Risueno *et al.* (2010) and a gene was tagged when expressed in phase or antiphase with DR5 oscillation.

### Plant material and growth conditions

All experiments were conducted with wild-type *Arabidopsis thaliana* (L.) Heyhn, accession Columbia-0 (Col-0). Seeds were surface sterilized and sown on half-strength Murashige and Skoog medium (Duchefa Biochemie B.V.) complemented with 1% (w/v) agarose and 1.5% (w/v) sucrose at pH 5.8. Seeds were stratified for at least 2 days at 4 °C. Seedlings were germinated in illuminated growth chambers under a 16h light/8h dark cycle (100 µmol m^-2^ s^-1^) at 21 °C. *N*-1-naphthylphthalamic acid (NPA) and NAA treatments and transcript level assays were as described by Himanen *et al.* (2002).

### Gene expression analysis

Total RNA from roots 5 days after germination was isolated with TRIzol reagent (Invitrogen), followed by treatment with RNase-free DNase I (Qiagen) according to the manufacturer’s instructions. The cDNA was prepared with the iScript™cDNA Synthesis Kit (Bio-Rad) from 1 μg of total RNA and 1:10 dilutions of total cDNA were used as template for quantitative RT-PCR. Genes and primers are listed in Supplementary Table 6. Means of samples were compared with two-way ANOVA (GraphPad Prism; V6.00, GraphPad Software).

### Statistical tests

Means of samples were compared with Student’s *t* test; equality between the population variances was assessed with the *F* test. Data were pooled from independent biological replicates unless specified otherwise.

## Results

### Identification of SSP genes in reference plant genomes

The authors searched for domains conserved across multiple plant species to identify potentially bioactive SSPs. Because the accuracy of gene models is crucial in this context, only species for which reliable genome annotations were available at the time this analysis was conducted were included: *Arabidopsis*, rice (*Oryza sativa*), poplar (*Populus trichocarpa*), grapevine (*Vitis vinifera*), and maize (*Zea mays*) (see Materials and Methods for details).

**Fig. 1.**
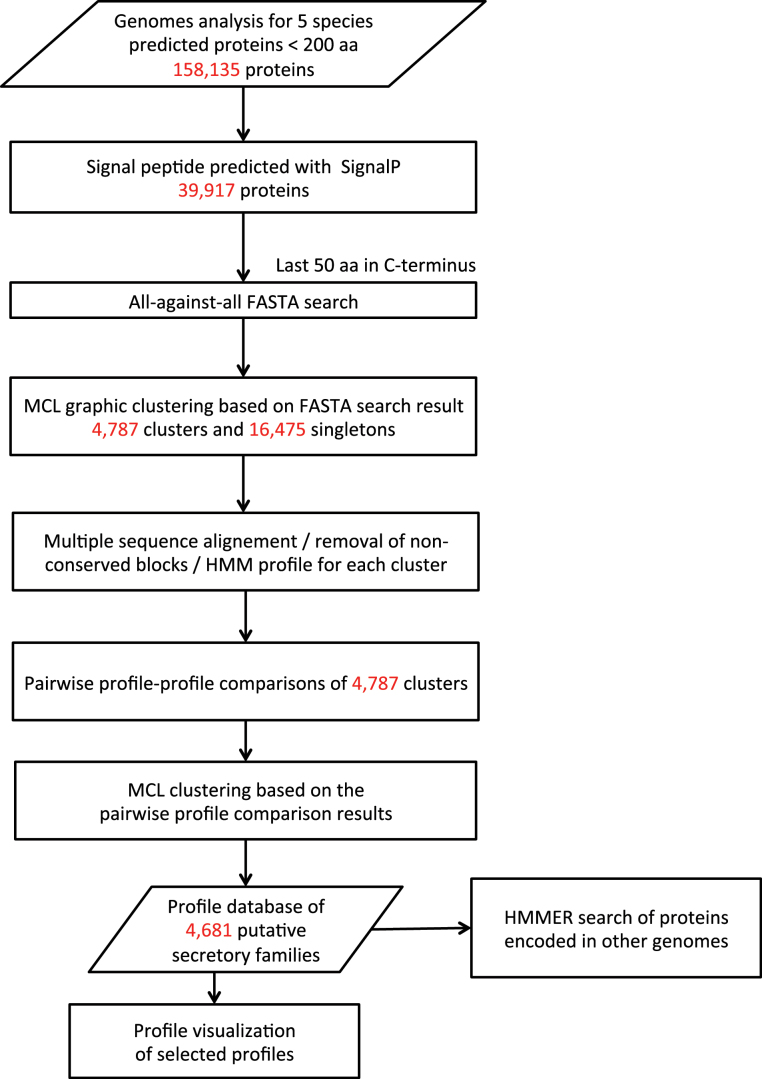
Flow chart of the pipeline for SSP family assembly. See Materials and Methods for details.

To benchmark SSP identification algorithms, the preproprotein primary sequences of signalling peptides known or suspected to be involved in root development (identified first in *Arabidopsis* in most cases) were collected. These include: CEP, CLAVATA3 (CLV3/CLE), GOLVEN/ROOT GROWTH FACTOR/CLE-LIKE (GLV/RGF/CLEL), IDA, PSK, PLANT PEPTIDE CONTAINING SULFATED TYROSINE (PSY), and additional cysteine-rich peptides (Table 1; Supplementary Table 3). In total, 195 *Arabidopsis* protein sequences were collected from these known secretory peptide families. Most of these short preproproteins contain an amino (N)-terminal signal peptide and a conserved carboxyl (C)-terminal end that is cleaved off to yield the mature signal. This latter sequence corresponds to the secreted bioactive portion of the peptide hormones shown in multiple cases to act as a ligand of leucine-rich repeat-receptor-like kinase (LRR-RLK) membrane proteins (Benková and Hejátko, 2009; Butenko *et al.*, 2009; Murphy *et al.*, 2012). The successive stages of the analytical pipeline aimed at identifying SSPs are explained below and summarized in Fig. 1.

**Table 1.** *Role of known plant secretory peptides in* Arabidopsis *root development*

Peptide family	Functions	Family ID^a^	*Arabidopsis* ^b^	Rice^d^	Poplar	Maize	Grapevine^e^	References
CLE	RAM maintenance, vascular development	f5, f9	30 (32)	45;38	48	44	1	Stahl *et al.* (2009); Kiyohara and Sawa (2012)
IDA	Lateral root emergence	f7	7 (6)	4;3	12	5	0	Kumpf *et al.* (2013)
PSK	QC cell division	f53	8 (6)	6;6	10	9	6	Heyman *et al.* (2013)
PSY	Cell elongation	f74, f4335	16 (3)	13;10	10	12	3	Amano *et al.* (2007)
RALF	Growth, rhizosphere acidification	f19, f839, f4248	40 (34)	19;24	23	30	9	Srivastava *et al.* (2009)
CEP1	Growth and branching	f195	6 (15^c^)	4;4	5	4	1	Delay *et al.* (2013*b* ); Roberts *et al.* (2013)
CEP2		f35	4	5;9	3	7	0	
GLV/RGF/CLEL	Lateral root formation, RAM maintenance, hair growth, gravitropism	f2	12	8;11	12	17	0	Matsuzaki *et al.* (2010); Whitford *et al.* (2012); Fernandez *et al.* (2013)
GASA	Gibberellic acid signalling, cell division (?)	f290	18 (15)	10;15	19	15	9	Roxrud *et al.* (2007)
f31	LR development	f31	4	6;2	5	5	0	This study; Hou *et al.* (2014); Vie *et al.* (2015)
f919	LR development	f919	2	3;2	1	9	0	This study
f1528	LR development	f1528	3	1;1	8	1	0	This study; Hou *et al.* (2014); Vie *et al.* (2015)

^a^ See Supplementary Table 2 for cluster [c#] and family [f#] content.

^b^ Number of previously described *Arabidopsis* peptides assembled in this study in the corresponding families. Peptides of the same family annotated in the *Arabidopsis* genome annotation TAIR10 are listed in parentheses.

^c^ Four CEP genes identified in the listed papers were not annotated in TAIR10. CEPs have been classified in a single family but the present study separates them into two families, in agreement with Roberts *et al.* (2013).

^d^ Two rice genome annotations provided complementary predicted SSPs: left numbers from RAP-DB, right from MSU6.1.

^e^ The grapevine genome codes for SSP gene families not represented in this table, i.e. marked as zero in the corresponding column. The discrepancy stems from the fact that these genes were not annotated in the grapevine genome version on which this study was based.

#### Length:

The average protein sequence length in the SSP benchmark set was 102 amino acids (Supplementary Table 3). The threshold of 200 amino acids was chosen as a conservative cut-off to exclude long protein sequences, resulting in 158,135 proteins selected from the predicted proteomes of the selected species (including splice variants). Approximately 24% of the predicted *Arabidopsis* proteins were shorter than 200 amino acids, yet the arbitrary protein sequence length cut-off removed only five out of 216 secretory peptides (2.3%) from the benchmark dataset [CEP (At1G31670), At3G50610, gibberellic acid-stimulated in *Arabidopsis* (GASA; At5G14920), putative precursor for endogenous peptide elicitor (PROPEP; At1G17750), and At1G73080].

#### Secretion:

Of these short proteins, 39,917 were predicted to contain an N-terminal hydrophobic region recognized as a cleavable signal sequence. However, not all characterized secretory signalling peptides carry such an identifiable sequence. Among the benchmark proteins, 40 (18.5%) did not contain a conventional signal peptide sequence, which may partly be explained by the arbitrary choice for the SignalP peptide identification parameters (Emanuelsson *et al.*, 2007).

#### Conserved C-terminal motif:

To reduce noise in sequence comparison, only the last 50 amino acids of the proteins were considered in the all-against-all FASTA sequence similarity search (e-value cut-off 10^–3^) (Pearson, 2000). The first round of aggregation with the MCL grouped 23,442 proteins into 4,787 clusters and left out 16,475 proteins as singletons.

### SSP family assembly

The candidate secretory peptides were further classified according to sequence homology by combining graphic clustering algorithms and pairwise profile comparisons (see Materials and Methods for details). To evaluate the performance of the clustering parameters, the assembly of the known *Arabidopsis* CLV3/CLE and GLV/RGF/CLEL secretory signalling peptides was examined. After the initial MCL analysis, yielding 4,787 independent clusters, the 32 CLE *Arabidopsis* proteins were still scattered in seven clusters (Supplementary Fig. 1) and the 11 *Arabidopsis* GLV proteins (including one splice variant) in five clusters (Supplementary Fig. 2).

The relationship between clusters was then calculated via pairwise profile comparisons and their higher-order relationship was determined with the MCL algorithm to aggregate related clusters into larger families whenever possible. The resulting clusters and aggregated families are numbered c# and f# as listed in Supplementary Table 2. The corresponding consensus and sequences can be searched online (http://bioinformatics.psb.ugent.be/webtools/PlantSSP/).

**Fig. 2.**
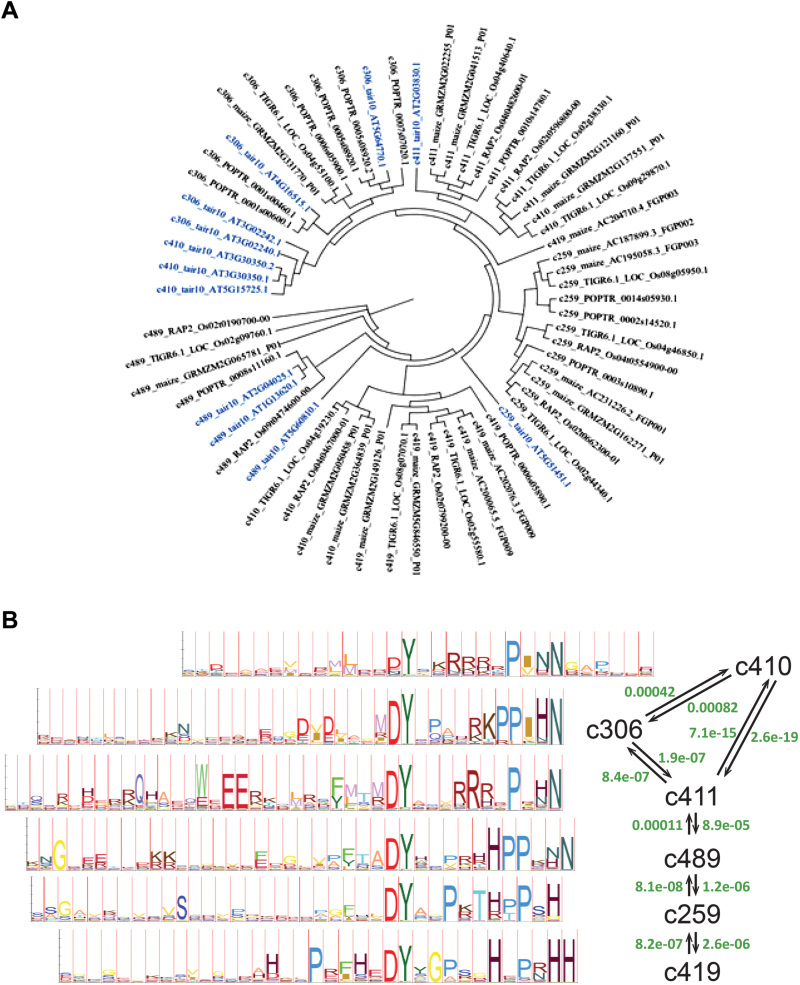
The GLV family identified via *de novo* global sequence comparison across reference species. (A) Phylogenetic tree of the GLV family across six plants’ genome annotation. Cluster IDs from the first MCL clustering are indicated in the first prefix of each protein sequence and the species ID (data source) corresponds to the second prefix. Known *Arabidopsis* GLV peptides are highlighted in blue. TAIR10, *Arabidopsis* TAIR10; RAP2, *Oryza sativa* RAP-DB, IRGSPbuild5; TIGR6.1, *O. sativa* MSU 6.1; PORTR: *Populus trichocarpa* JGI v156; vitis: *Vitis vinifera*, Genoscope v1; maize: *Zea mays* ZmB73_5a. (B) GLV/RGF/CLEL cluster relationships. Black lines represent the connectivity between GLV clusters and green numbers indicate the e-value of HMM profile similarity resulting from pairwise cluster comparisons (see Supplementary Table 2 for cluster [c#] and family [f#] content).

The MCL clustering based on the protein profiles markedly improved the resolution of known secretory families. For example, the *Arabidopsis* GLV peptides were all grouped in a single family (Fig. 2A; Supplementary Fig. 2; Table 1; Supplementary Table 2). As expected, the topology of the cluster connectivity network built with the predicted proteins selected from the five reference species resembles the phylogenetic relationships between peptides in the family, as close sequences according to the phylogenetic tree tend to group together in the same cluster or in neighbouring clusters (Fig. 2B).

The assembly of the large CLE peptide family further illustrates the usefulness of the sequence clustering method used. A classical multiple sequence alignment of the CLE peptides identified conserved amino acid positions (Supplementary Fig. 3). In comparison, in the analytical pipeline, the TribeMCL clustering based on the FASTA search data (which removes non-aligned gaps and non-conserved positions) first grouped CLE peptides with the most similar bioactive domains, resulting in seven clusters (Supplementary Fig. 1). Next, a HMM was built to represent each cluster separately and the second round of TribeMCL clustering resolved the cluster relationship into two families (Supplementary Fig. 1, inset), which coincidentally correspond to the subgroups involved in either root apical meristem (RAM) maintenance or vascular development (Kiyohara and Sawa, 2012).

In summary, the multispecies genome-scale analytical pipeline can reconstruct known secretory peptide families and distinguish subfunctional classes without prior knowledge of specific sequences, but simply taking into consideration the preproprotein length, the presence of a N-terminal signal sequence and the conservation of C-terminal oligopeptides.

**Fig. 3.**
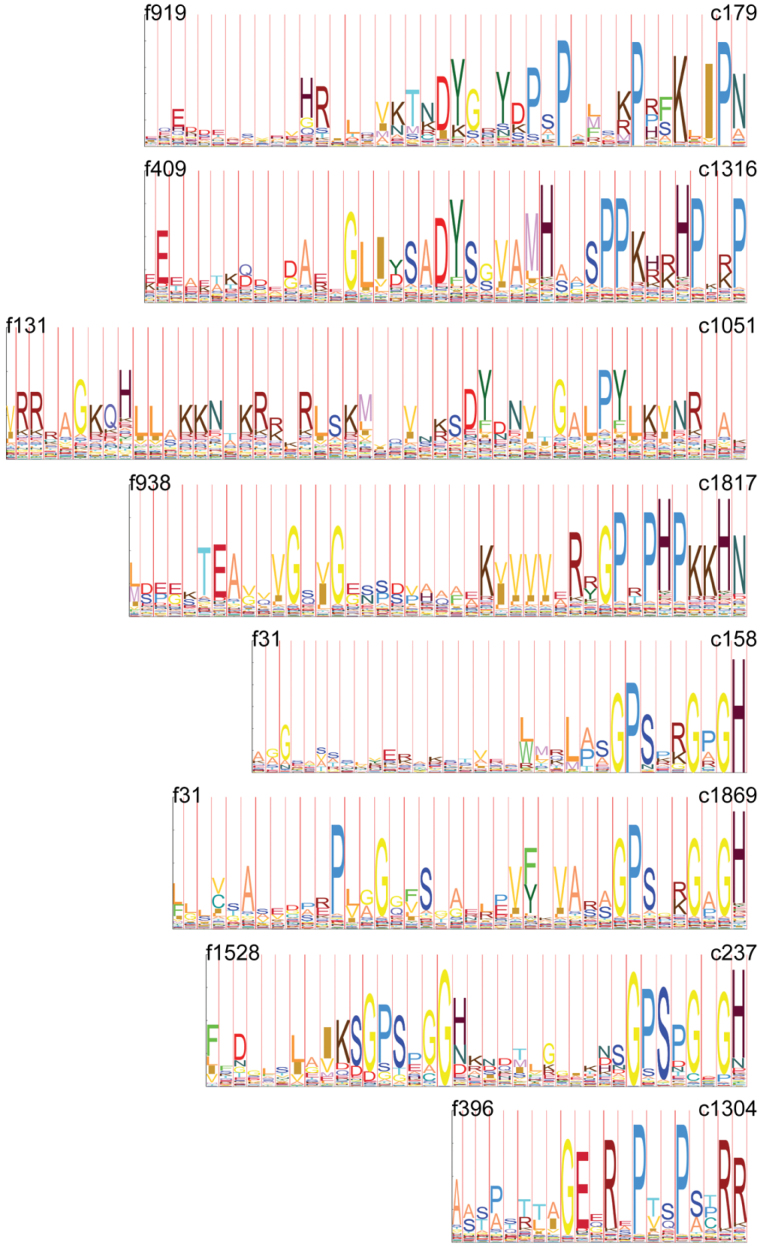
Conserved SSP C-terminal sequences. Consensus sequences are represented for previously uncharacterized families. Conserved protein residues are higher in the HMM profile (see Supplementary Table 2 for cluster [c#] and family [f#] content).

In addition, the manual curation of previously unreported consensus sequences revealed conspicuous patterns commonly observed in known signalling peptide families. For example, a tyrosine residue was found in the conserved motif in multiple families (e.g., f131, f409, f919; Fig. 3). Such a tyrosine residue is known to be sulfated in the GLV, PSK, and PSY mature signalling peptides, where it is also preceded by an aspartic acid residue. Its presence and its post-translational modification are crucial for bioactivity (Komori *et al.*, 2009; Matsuzaki *et al.*, 2010; Whitford *et al.*, 2012). The conserved motifs often end at or very near the last C-terminal residue of the precursor protein and contain one or several proline residues that might act as hinges when the peptide ligand binds to its receptor (Fig. 3). Together, these observations indicate that the global *de novo* sequence search method used in this study provides valuable hints about unrecognized *bona fide* SSPs.

### Secretory peptide evolution in plants

On the basis of the SSP library created with the five reference species, the SSP family content was extended to 32 publicly available genomes of photosynthetic organisms (Supplementary Table 1) filtered with the same method as for the initial clustering. The resulting secretory peptide family library is a useful resource to search for known, as well as uncharacterized, SSPs encoded in plant genomes (http://bioinformatics.psb.ugent.be/webtools/PlantSSP/).

**Fig. 4.**
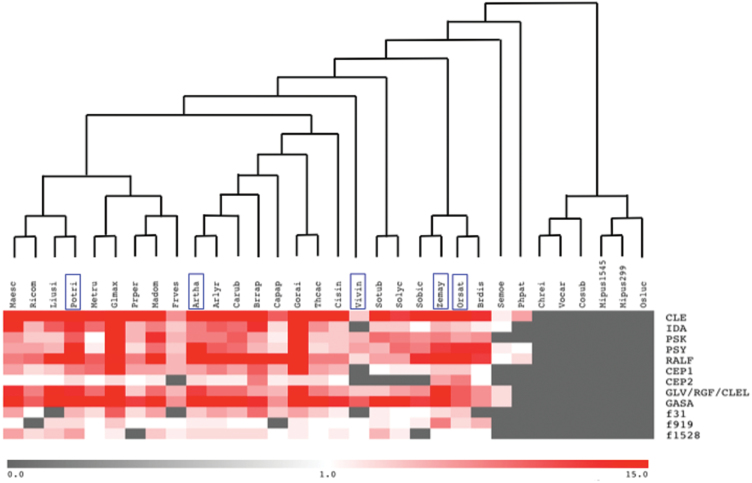
SSP evolution in plants. For each genome, the number of proteins in a given secretory peptide family is represented as shown in the bottom bar: species with no SSP are encoded in grey, those with one SSP in white, and those with higher number of SSPs in increasingly deep red. The graph was generated with the MeV software package (Saeed *et al.*, 2003). Blue boxes indicate five reference species. Arlyr: *Arabidopsis lyrata*; Artha: *Arabidopsis thaliana*; Brdis: *Brachypodium distachyon*; Brrap: *Brassica rapa*; Capap: *Carica papaya*; Carub: *Capsella rubella*; Chrei: *Chlamydomonas reinhardtii*; Cisin: *Citrus sinensis*; Cosub: *Coccomyxa subellipsoidea*; Frves: *Fragaria vesca*; Glmax: *Glycine max*; Gorai: *Gossypium raimondii*; Liusi: *Linum usitatissimum*; Madom: *Malus domestica*; Maesc: *Manihot esculenta*; Metru: *Medicago truncatula*; Mipus1545: *Micromonas pusilla* CCMP1545; Mipus299: *M. pusilla RCC299*; Orsat: *Oryza sativa*; Osluc: *Ostreococcus lucimarinus*; Phpat: *Physcomitrella patens*; Potri: *Populus trichocarpa*; Prper: *Prunus persica*; Ricom: *Ricinus communis*; Semoe: *Selaginella moellendorffii*; Sobic: *Sorghum bicolor*; Solyc: *Solanum lycopersicum*; Sotub: *Solanum tuberosum*; Thcac: *Theobroma cacao*; Vivin: *Vitis vinifera*; Vocar: *Volvox carteri*; Zemay: *Zea mays*. See Supplementary Table 1 for genome information and Supplementary Table 4 for family content and gene ID.

Despite the challenge of short ORF prediction and the unequal quality of genome annotations, a clear trend of SSP expansion can be observed: known SSPs are encoded in large families in land plants but are almost completely absent in Chlorophyta (Fig. 4). This phylogenetic pattern may reflect that unknown sets of intercellular signals, among which secretory peptides, were required for the development of complex architectures characterizing the land plant lineage.

### SSP gene regulation in the course of *Arabidopsis* root development

Considering the established role of several secretory peptides in root development, the authors examined how SSP genes were expressed during LR formation in *Arabidopsis*. The aim was to test whether the spatiotemporal specificity of their transcription pattern could be a valuable predictor for their possible involvement in root development. To this end, SSP transcript levels were analysed in transcriptome experiments addressing early aspects of LR initiation, which takes place in the pericycle associated with the xylem poles and depends on a SOLITARY ROOT/INDOLE-3-ACETIC ACID14 (SLR/IAA14)-mediated auxin signalling cascade. Three datasets follow the transcriptional regulation occurring during the induction of LR initiation upon treatment: (i) with auxin and depending on SLR/IAA14 (Vanneste *et al.*, 2005); (ii) with auxin and naxillin, a non-auxin-like LR-inducing molecule (De Rybel *et al.*, 2012); and (iii) with auxin, specifically changes in the pericycle cells at the xylem pole (De Smet *et al.*, 2008). Two other datasets address the spatial expression pattern of genes: (iv) the differential between the pericycle cells at the xylem or phloem pole (Parizot *et al.*, 2012); and (v) specificity in the LRP, either in the entire pericycle or in one of its subpopulations (xylem or phloem pole) (Brady *et al.*, 2007). The last dataset (vi) focuses on the temporal expression pattern in phase or antiphase with the auxin transcriptional response marker DR5 in the basal meristem (Moreno-Risueno *et al.*, 2010).

First, the transcriptomics data were searched for patterns associated with known SSP gene families (Table 1). Although a portion of the SSP sequences are not represented on the Affymetrix ATH1 microarray (65 out of 148; 44%), half of the 83 known SSP genes with a corresponding probeset had a specific spatiotemporal expression pattern in a least one of the analysed experiments (FC ≥ 1.5, *P* ≤ 0.01; for additional information, see Materials and Methods; see also Supplementary Table 4). This observation suggests that many more secretory peptides might be involved in apoplastic signalling during LR initiation than previously recognized.

This analysis was extended to genes belonging to uncharacterized SSP families, coding for motifs reminiscent of known signalling peptides (Fig. 3), and represented on the ATH1 microarray. Five genes in three families showed significant changes in at least one of the analysed experiments according to the same criteria as above (Table 2). At4G37295, At4G34600, and At4G37290 are induced in the xylem pole pericycle upon auxin treatment and depend on the IAA14/SLR pathway. At4G37295 and At4G37290 are also induced upon naxillin treatment. At4G37295 is specifically expressed in the LRP. At4G28460 and At1G49800 are in phase with the oscillating auxin response observed in the basal meristem with the DR5 marker, and the expression of At4G28460 is also higher in the phloem pole pericycle than that in the xylem pole pericycle. In conclusion, the expression of a large fraction of SSP-encoding genes is regulated during LR initiation, whether they have been recognized previously as involved in development or not.

**Table 2.** *Specific spatiotemporal expression of uncharacterized SSP genes during lateral root initiation (based on public transcriptome data*)

Characteristics	f31-1^a^	f31-2	f31-3	f919-2	f1528-1	f1528-2	f1528-3
AGI ID	AT3G06090	AT4G37295	AT4G28460	AT4G34600	AT1G49800	AT2G23270	AT4G37290
ATH1 probe set	256391_at	253047_at	253796_at	253246_at	259809_at	245082_at	253044_at
***SLR-dependent auxin pathway***							
Auxin induction^a^		0–2 h		2–6 h			0–2 h
SLR dependence		Yes		Yes			Yes
***Auxin and naxillin induction***							
Auxin induction^b^		0–2 h		0–2 h		0–2 h	0–2 h
Naxillin induction^b^		0–6 h					0–2 h
***Xylem pole pericycle***							
Auxin induction^b^		0–2 h		2–6 h			0–2 h
Pericycle differential expression			PPP				
Radial layers		Primordium					
DR5 oscillation^c^			P2		P5		

^a^ The first number indicates the identified family number. Corresponds with family names in Table 1.

^b^ Time after treatment: between 0 and 2h (early transition), 2 and 6h (late transition), or 0 and 6h (slow transition).

^c^ Px indicates a cluster in phase with DR5 oscillations.

PPP, phloem pole pericycle layer.

### SSP functional analysis

The activity of SSPs can be tested by the application of chemically synthesized peptides on plant tissues because the response they induce often mimics the cognate genetic gain-of-function phenotypes, as shown in *Arabidopsis* roots (Fernandez *et al.*, 2013; Fiers *et al.*, 2006; Matsuzaki *et al.*, 2010; Whitford *et al.*, 2012). Such experiments demonstrated that the bioactive portion of the SSP preproproteins is encoded in their C-terminal conserved sequences.

**Fig. 5.**
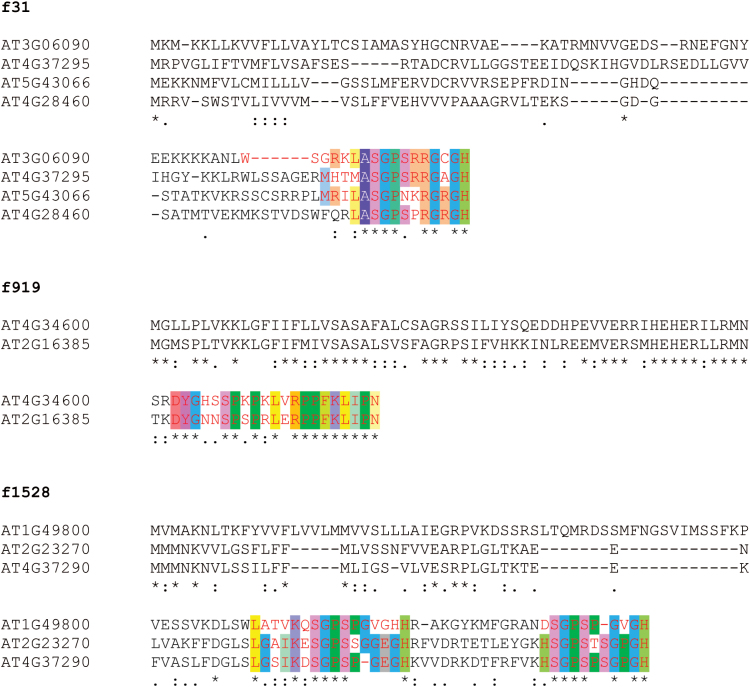
Primary sequence alignment of *Arabidopsis* SSPs tested in root development assays. The multiple sequence alignment was generated with ClustalW2. f# refers to SSP families as defined in Supplementary Table 2.

To investigate the potential role of uncharacterized SSPs, seedlings were grown on agar medium supplemented with synthetic peptides corresponding to conserved C-terminal stretches (Fig. 5; Supplementary Table 5). Whereas synthetic SSPs, including members of the CLV3/CLE and GLV/RGF/CLEL families, are active at nanomolar concentrations (Murphy *et al.*, 2012), the absence of certain post-translational modifications in synthetic copies has been shown to reduce bioactivity compared with native peptides (Matsubayashi, 2014; Seitz, 2000; Shinohara and Matsubayashi, 2013). To avoid false-negative results due to lack of post-translational modification, micromolar concentrations of synthetic peptides were applied, as is commonly reported in such experiments.

**Fig. 6.**
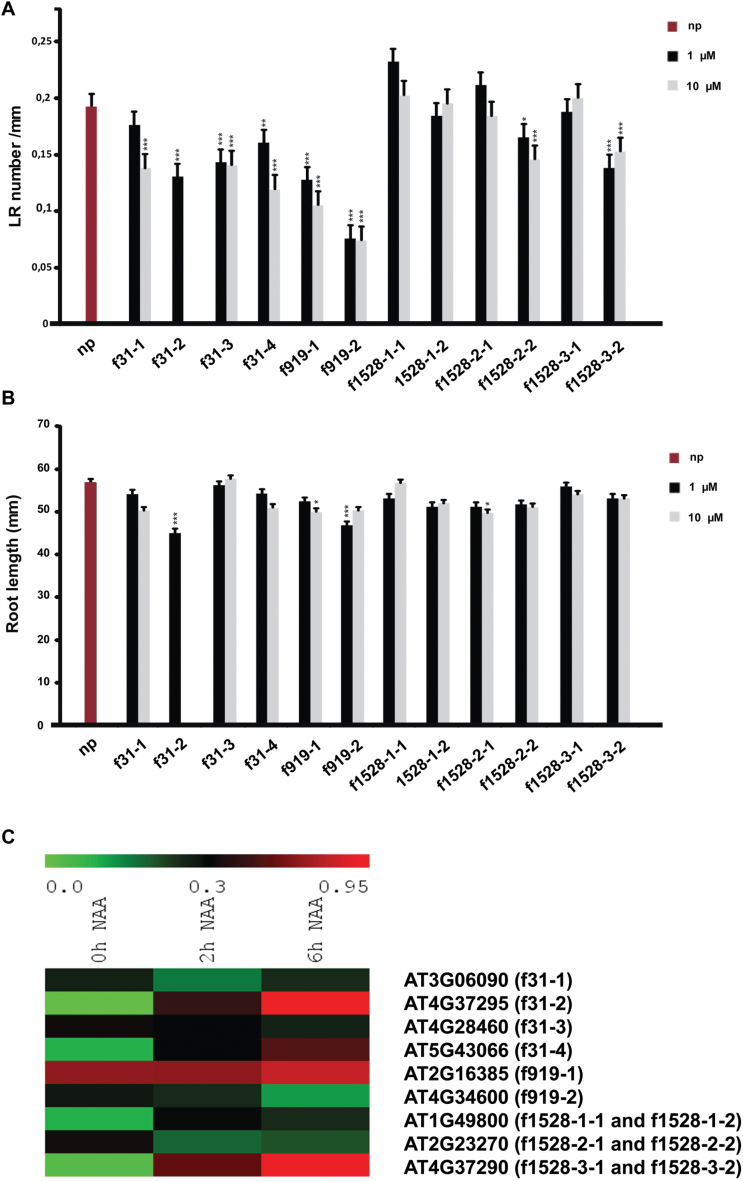
Root-related phenotypes induced by the identified SSPs. (A) Number of emerged LRs per unit length (mm) (*n* = 20–37). (B) Primary root length (*n* = 19–44). Seedlings (10 days after germination) were compared with controls after treatment with the indicated peptides. Error bars represent the 95% confidence interval. Asterisks mark significant differences: * *P* < 0.05; ** *P* < 0.005, *** *P* < 0.001. Data were pooled from independent biological replicates. (C) Induction of SSP gene transcription by auxin. Seedlings were treated with 1 µM NAA for the indicated time points. Fold changes were measured after qRT-PCR analysis of root tissues. Data are shown for one of two independent experiments. np, no peptide.

The number of LRs and the primary root length were compared between control seedlings and seedlings treated with 1 µM or 10 µM of peptides for three uncharacterized families. Peptides (Pep) from families f31 and f919 decreased the number of emerged LRs. Pep f919-2 (At4G34600), in particular, resulted in a 70% decrease compared with control untreated seedlings (Fig. 6A; Supplementary Fig. 4). In all cases, the effect was stronger or only detectable at 10 µM. Furthermore, plantlets treated with 10 µM of Pep f31-2 (At4G37295) were pale and arrested in growth. From the family f1528, only Pep f1528-2-2 (At2G23270) and Pep f1528-3-2 (At4G37290) induced significant differences compared with control untreated plants (Fig. 6A; Supplementary Fig. 4). Peptides inhibiting LR emergence had no detectable effect on primary root growth, except Pep f31-1 and Pep f919-2 and, at high concentration, Pep f919-1 and Pep f1528-2-1 (Fig. 6B; Supplementary Fig. 4). As expected, treatment with randomized Pep f31-2 and Pep f919-2 showed no effect on either root growth or LR emergence.

In a recent independent study, Hou *et al.* (2014) showed that genes coding for peptides secreted in the apoplast are induced by pathogen-associated molecular patterns (PAMPs) and amplify immunity. The so-called PAMP-induced peptides PIP1 and PIP2 correspond to Pep f31-3 and Pep f1528-2, respectively, and share a SGPS motif in their C-terminal conserved region. The same report showed that the overexpression of *prePIP1* and *prePIP2* and the application of PIP1 and PIP2 synthetic peptides inhibited root growth, in agreement with the present results. The PIP family was further extended to include PIP-LIKE (PIPL) peptides, related to IDA/IDL and CEP peptides, and possibly involved in the response to biotic and abiotic stresses (Vie *et al.*, 2015).

To confirm the plausible role of the corresponding SPP genes in LR development, the authors quantified their transcriptional changes in the LR-inducible system (Himanen *et al.*, 2002). In this experimental set-up, the first formative divisions are prevented by the auxin transport inhibitor NPA. Later, upon auxin (NAA) treatment, cells in the pericycle layer engage actively and synchronously in division. Quantitative reverse-transcription PCR (qRT-PCR) analysis showed very specific transcription patterns for some candidates (Fig. 6C).

Expression of the genes analysed increased after both 2h and 6h for AT4G37295, AT5G43066, AT4G37290, and AT2G16385, but continuously decreased for AT4G28460 and AT4G34600. The expression level of AT3G06090 and AT2G23270 decreased after 2h and increased after 6h, while AT1G49800 had the opposite pattern of expression. These changes are in accordance with the transcriptome data and further indicate that the tested genes are involved in root development, including LR initiation (Fernandez *et al.*, 2013; Ohyama *et al.*, 2008).

**Fig. 7.**
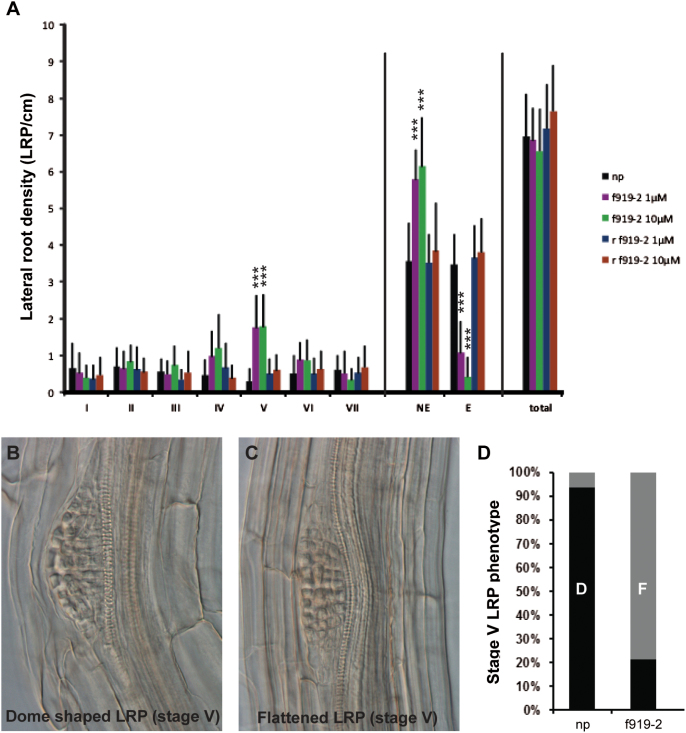
LR-related phenotypes induced by the f919-2 peptide. (A) Distribution of LR developmental stages in roots 12 days after germination. I–VII, primordium stages; NE, non-emerged primordia; E, emerged LRs; total, total number of LRs; np, no peptide; r, randomized peptide. Results for one of two independent experiments are shown (see Materials and Methods). Error bars represent the 95% confidence interval. Asterisks indicate significant differences compared with the no-peptide control (*** *P* < 0.001). (B, C) Differential interference contrast images of representative stage V LRP-treated (C) or not treated (B) with the f919-2 peptide (10 μM). (D) Relative distribution of the normal and flattened stage V LRP. D, dome-shaped primordia (black); F, flattened primordia (grey) (*n* = 15–48).

Finally, the authors investigated whether the phenotype caused by newly discovered bioactive peptides may be an indication of their plausible function. Cleared roots were analysed after treatment with Pep f919-2, which is the strongest inhibitor of root branching in this study (Fig. 6), and compared with untreated roots or roots treated with a randomized Pep f919-2 (Fig. 7). This experiment confirmed that Pep f919-2 significantly decreased the number of emerged LRs. However, the peptide treatment did not affect the number of primordia being initiated (Fig. 7A). Instead, Pep f919-2-treated roots carried an unusually high number of primordia at stage V of development, which normally precedes the progression of the LR through the overlying cell layers (endodermis, cortex and epidermis) before it emerges from the body of the main root (Malamy and Benfey, 1997). Furthermore, the shape of the primordia was clearly different depending on the root treatment. Most primordia grew with a classical dome shape in the control plants (Fig. 7B). In contrast, in Pep f919-2-treated roots, the vast majority of LRPs appeared flattened as if pressed against the overlying tissues (Fig. 7C, D).

The reduced LR density and flattened primordium phenotypes are very similar to those of the *ida* and *hae hsl2* mutants (Kumpf *et al.*, 2013). In wild-type roots, LR emergence is promoted by auxin fluxes redirected in the LRP and surrounding tissues that eventually lead to the induction of auxin- and IDA-responsive genes. These genes code for cell wall-remodelling enzymes that trigger cell separation as they open the way to the protruding primordium (reviewed in Atkinson *et al.*, 2014). In *ida* and *hae hsl2* as well as in other auxin transporter mutants, overlying tissues fail to soften and LRP development stalls as emergence is blocked.

These observations suggest that AT4G34600 takes part in the events preparing for the penetration of the LR through the outer layers of the root: its expression normally decreases during LR formation, and the exogenous application of the f919-2 secreted peptide it encodes resulted in compression of the LRP and the inhibition of LR emergence. While the molecular function of AT4G34600 remains to be elucidated, the data collected so far provide a good framework for future studies.

## Discussion

A bottleneck in the functional study of signalling peptides in plant growth and development has been the identification of the encoding genes. Whereas the sequencing of different plant genomes has led to the prediction of numerous small genes, some of which potentially encode signalling peptides, the identification of conserved families via comparative genomics is difficult, because their bioactive domains are restricted to just a few amino acids.

Unlike previous studies solely relying on the SSP information embedded in the *Arabidopsis* genome annotation (Lease and Walker, 2006; Silverstein *et al.*, 2007), the *de novo* comparative genomics approach used in this study takes advantage of additional available plant genomes without a prior knowledge of the SSP sequence information, resulting in the fine resolution of the SSP families. The presence of multiple plant species in the analytical pipeline increases the sensitivity to separate large SSP families into multiple smaller groups. The subsequent profile comparison improved the clustering specificity. The authors’ bioinformatic approach produced a classification that can be updated rapidly and regularly as genome annotation information accrues. The searchable public website presenting the SSP classes and the corresponding consensus sequences across multiple plant species is a valuable resource to explore understudied peptide regulators or to identify homologues in crops (http://bioinformatics.psb.ugent.be/webtools/PlantSSP/). Finally, the consensus motifs that were found can serve as functional domain hallmarks to search for small missed genes, either in assembled genome sequences or in shorter RNA-sequence reads.

The meta-analysis of transcriptome data linked to LR development (Parizot *et al.*, 2010) has already led to the discovery of several genes proven to be involved in LR development in follow-up genetic studies (GATA23, De Rybel *et al.*, 2010; E2Fa, Berckmans *et al.*, 2011; PdBG1, Benitez-Alfonso *et al.*, 2013; totipotency genes, Chupeau *et al.*, 2013; PLT3, Zhang *et al.*, 2013; PDCB1, Maule *et al.*, 2013). To point out the potential involvement of unidentified candidate SSP families in the process of LR development, the authors of the present study identified genes with specific expression patterns during LR initiation and showed that the majority of encoded conserved peptides tested altered the growth of *Arabidopsis* roots when applied exogenously, some in very specific ways. Peptide assays are cheap, easy, and rapid first steps toward the classification of non-cell-autonomous factors potentially involved in development. They can be adapted to a wide range of processes.

Of course, the refined understanding of the SSP function requires additional studies to avoid the pitfalls of gain-of-function phenotypes: non-physiological concentrations of signal molecules may create artefacts, for example, by hijacking downstream pathways of related, but distinct, peptide signal(s); in addition, exogenous applications are not directional, whereas SSP genes are often expressed in very specific cell types, as again demonstrated here. Nevertheless, these results indicate that the successive combination of SSP gene annotation, expression studies, and *in vivo* peptide assays is a useful approach to start rapidly probing the complexity of the extracellular signalling networks that drive plant tissue growth and development.

## Supplementary data

Supplementary data are available at *JXB* online.

Supplementary Fig. 1. CLE peptide bioactive domain defined by multiple sequence alignments and HMM logos.

Supplementary Fig. 2. GLV peptide bioactive domain defined by multiple sequence alignments.

Supplementary Fig. 3. Multiple sequence alignment of the C-terminal 50 amino acids of the *Arabidopsis* CLE family.

Supplementary Fig. 4. Root-related phenotypes are not induced by randomized peptide sequences.

Supplementary Table 1. Genomes of photosynthetic organisms included in the SSP family definition.

Supplementary Table 2. SSP clusters and families constructed with the Markov Cluster Algorithm and Profile Comparer and based on the five reference species.

Supplementary Table 3. SSP genes collected as a benchmark set for *de novo* secretory peptide detection algorithms.

Supplementary Table 4. Specific expression patterns of known SSP genes during LR formation.

Supplementary Table 5. Synthetic peptide sequences tested for effect on root growth and development.

Supplementary Table 6. Primers used for qRT-PCR analysis.

Supplementary Data

## Abbreviations:

ANOVA: analysis of variance
CEP1: C-TERMINALLY ENCODED PEPTIDE 1
CLV3/CLE: CLAVATA3
FC: fold change
GLV: *GOLVEN* gene
GLV/RGF/CLEL: GOLVEN/ROOT GROWTH FACTOR/CLE-LIKE
HAE: HAESA
HMM: hidden Markov model
HSL2: HAESA-Like 2
IDA: INFLORESCENCE DEFICIENT IN ABCISSION
LR: lateral root
LRP: lateral root primordium
LRR-RLK: leucine-rich-repeat receptor-like kinase
MCL: Markov Cluster Algorithm
NAA: 1-naphthaleneacetic acid
NPA: *N*-1-naphthylphthalamic acid
ORF: open reading frame
PAMP: pathogen-associated molecular pattern
Pep: peptide
PRC: Profile Comparer
PSK: PHYTOSULFOKINE
PSY: PLANT PEPTIDE CONTAINING SULFATED TYROSINE
QC: quiescent centre
qRT-PCR: quantitative reverse-transcription PCR
RAM: root apical meristem
SLR/IAA14: SOLITARY ROOT/INDOLE-3-ACETIC ACID14
SSP: small secretory peptide.

## References

[CIT0001] Amano Y Tsubouchi H Shinohara H Ogawa M Matsubayashi Y 2007 Tyrosine-sulfated glycopeptide involved in cellular proliferation and expansion in *Arabidopsis* . Proceedings of the National Academy of Sciences of the United States of America 104, 18333–18338. 17989228 10.1073/pnas.0706403104 PMC2084343

[CIT0002] Atkinson JA Rasmussen A Traini R Voß U Sturrock C Mooney SJ Wells DM Bennett MJ 2014 Branching out in roots: uncovering form, function, and regulation. Plant Physiology 166, 538–550. 25136060 10.1104/pp.114.245423 PMC4213086

[CIT0003] Bendtsen JD Nielsen H von Heijne G Brunak S 2004 Improved prediction of signal peptides: SignalP 3.0. Journal of Molecular Biology 340, 783–795. 15223320 10.1016/j.jmb.2004.05.028

[CIT0004] Benitez-Alfonso Y Faulkner C Pendle A Miyashima S Helariutta Y Maule A 2013 Symplastic intercellular connectivity regulates lateral root patterning. Developmental Cell 26, 136–147. 23850190 10.1016/j.devcel.2013.06.010

[CIT0005] Benková E Hejátko J 2009 Hormone interactions at the root apical meristem. Plant Molecular Biology 69, 383–396. 18807199 10.1007/s11103-008-9393-6

[CIT0006] Berckmans B Vassileva V Schmid SPC 2011 Auxin-dependent cell cycle reactivation through transcriptional regulation of *Arabidopsis E2Fa* by lateral organ boundary proteins. The Plant Cell 23, 3671–3683. 22003076 10.1105/tpc.111.088377 PMC3229142

[CIT0007] Brady SM Orlando DA Lee J-Y Wang JY Koch J Dinneny JR Mace D Ohler U Benfey PN 2007 A high-resolution root spatiotemporal map reveals dominant expression patterns. Science 318, 801–806. 17975066 10.1126/science.1146265

[CIT0008] Butenko MA Vie AK Brembu T Aalen RB Bones AM 2009 Plant peptides in signalling: looking for new partners. Trends in Plant Science 14, 255–263. 19362511 10.1016/j.tplants.2009.02.002

[CIT0009] Chupeau M-C Granier F Pichon O Renou J-P Gaudin V Chupeau Y 2013 Characterization of the early events leading to totipotency in an *Arabidopsis* protoplast liquid culture by temporal transcript profiling. The Plant Cell 25, 2444–2463. 23903317 10.1105/tpc.113.109538 PMC3753376

[CIT0010] De Rybel B Audenaert D Xuan W 2012 A role for the root cap in root branching revealed by the non-auxin probe naxillin. Nature Chemical Biology 8, 798–805. 22885787 10.1038/nchembio.1044 PMC3735367

[CIT0011] De Rybel B Vassileva V Parizot B 2010 A novel Aux/IAA28 signaling cascade activates GATA23-dependent specification of lateral root founder cell identity. Current Biology 20, 1697–1706. 20888232 10.1016/j.cub.2010.09.007

[CIT0012] De Smet I Vassileva V De Rybel B 2008 Receptor-like kinase ACR4 restricts formative cell divisions in the Arabidopsis root. Science 322, 594–597. 18948541 10.1126/science.1160158

[CIT0013] Delay C Imin N Djordjevic MA 2013 *a* Regulation of *Arabidopsis* root development by small signaling peptides. Frontiers in Plant Science 4, 352. 24046775 10.3389/fpls.2013.00352 PMC3764427

[CIT0014] Delay C Imin N Djordjevic MA 2013 *b* *CEP* genes regulate root and shoot development in response to environmental cues and are specific to seed plants. Journal of Experimental Botany 64, 5383–5394. 24179096 10.1093/jxb/ert332

[CIT0015] Edgar RC 2004 MUSCLE: a multiple sequence alignment with reduced time and space complexity. BMC Bioinformatics 5, 113. 15318951 10.1186/1471-2105-5-113 PMC517706

[CIT0016] Emanuelsson O Brunak S von Heijne G Nielsen H 2007 Locating proteins in the cell using TargetP, SignalP and related tools. Nature Protocols 2, 953–971. 17446895 10.1038/nprot.2007.131

[CIT0017] Enright AJ Van Dongen S Ouzounis CA 2002 An efficient algorithm for large-scale detection of protein families. Nucleic Acids Research 30, 1575–1584. 11917018 10.1093/nar/30.7.1575 PMC101833

[CIT0018] Fernandez A Drozdzecki A Hoogewijs K Nguyen A Beeckman T Madder A Hilson P 2013 Transcriptional and functional classification of the GOLVEN/ROOT GROWTH FACTOR/CLE-like signaling peptides reveals their role in lateral root and hair formation. Plant Physiology 161, 954–970. 23370719 10.1104/pp.112.206029 PMC3561032

[CIT0019] Fiers M Golemiec E van der Schors R van der Geest L Li KW Stiekema WJ Liu C-M 2006 The CLAVATA3/ESR motif of CLAVATA3 is functionally independent from the nonconserved flanking sequences. Plant Physiology 141, 1284–1292. 16751438 10.1104/pp.106.080671 PMC1533954

[CIT0020] Grienenberger E Fletcher JC 2015 Polypeptide signaling molecules in plant development. Current Opinion in Plant Biology 23C, 8–14. 25449721 10.1016/j.pbi.2014.09.013

[CIT0021] Guyomarc’h S Lucas M Laplaze L 2010 Lateral/secondary roots. In: Encyclopedia of Life Sciences (eLS). Chichester: John Wiley & Sons. doi: 10.1002/9780470015902.a0002060.pub2

[CIT0022] Hanada K Higuchi-Takeuchi M Okamoto M 2013 Small open reading frames associated with morphogenesis are hidden in plant genomes. Proceedings of the National Academy of Sciences of the United States of America 110, 2395–2400. 23341627 10.1073/pnas.1213958110 PMC3568369

[CIT0023] Heyman J Cools T Vandenbussche F 2013 ERF115 controls root quiescent center cell division and stem cell replenishment. Science 342, 860–863. 24158907 10.1126/science.1240667

[CIT0024] Himanen K Boucheron E Vanneste S de Almeida Engler J Inzé D Beeckman T 2002 Auxin-mediated cell cycle activation during early lateral root initiation. The Plant Cell 14, 2339–2351. 12368490 10.1105/tpc.004960 PMC151221

[CIT0025] Hou S Wang X Chen D Yang X Wang M Turrà D Di Pietro A Zhang W 2014 The secreted peptide PIP1 amplifies immunity through receptor-like kinase 7. PLoS Pathogens 10, e1004331. 25188390 10.1371/journal.ppat.1004331 PMC4154866

[CIT0026] Imin N Mohd-Radzman NA Ogilvie HA Djordjevic MA 2013 The peptide-encoding *CEP1* gene modulates lateral root and nodule numbers in *Medicago truncatula* . Journal of Experimental Botany 64, 5395–5409. 24259455 10.1093/jxb/ert369

[CIT0027] Irizarry RA Hobbs B Collin F Beazer-Barclay YD Antonellis KJ Scherf U Speed TP 2003 Exploration, normalization, and summaries of high density oligonucleotide array probe level data. Biostatistics 4, 249–264. 12925520 10.1093/biostatistics/4.2.249

[CIT0028] Jaillon O Aury J-M Noel B 2007 The grapevine genome sequence suggests ancestral hexaploidization in major angiosperm phyla. Nature 449, 463–467. 17721507 10.1038/nature06148

[CIT0029] Kawahara Y de la Bastide M Hamilton JP 2013 Improvement of the *Oryza sativa* Nipponbare reference genome using next generation sequence and optical map data. Rice 6, 4. 24280374 10.1186/1939-8433-6-4 PMC5395016

[CIT0030] Kiyohara S Sawa S 2012 CLE signaling systems during plant development and nematode infection. Plant & Cell Physiology 53, 1989–1999. 23045524 10.1093/pcp/pcs136

[CIT0031] Komori R Amano Y Ogawa-Ohnishi M Matsubayashi Y 2009 Identification of tyrosylprotein sulfotransferase in *Arabidopsis* . Proceedings of the National Academy of Sciences of the United States of America 106, 15067–15072. 19666544 10.1073/pnas.0902801106 PMC2736448

[CIT0032] Kumpf RP Shi C-L Larrieu A Stø IM Butenko MA Péret B Riiser ES Bennett MJ Aalen RB 2013 Floral organ abscission peptide IDA and its HAE/HSL2 receptors control cell separation during lateral root emergence. Proceedings of the National Academy of Sciences of the United States of America 110, 5235–5240. 23479623 10.1073/pnas.1210835110 PMC3612645

[CIT0033] Lease KA Walker JC 2006 The Arabidopsis unannotated secreted peptide database, a resource for plant peptidomics. Plant Physiology 142, 831–838. 16998087 10.1104/pp.106.086041 PMC1630735

[CIT0034] Lease KA Walker JC 2010 Bioinformatic identification of plant peptides. Methods in Molecular Biology 615, 375–383. 20013221 10.1007/978-1-60761-535-4_26

[CIT0035] Levesque MP Vernoux T Busch W 2006 Whole-genome analysis of the SHORT-ROOT developmental pathway in *Arabidopsis* . PLoS Biology 4, e143. 16640459 10.1371/journal.pbio.0040143 PMC1450008

[CIT0036] Madera M 2008 Profile Comparer: a program for scoring and aligning profile hidden Markov models. Bioinformatics 24, 2630–2631. 18845584 10.1093/bioinformatics/btn504 PMC2579712

[CIT0037] Malamy JE Benfey PN 1997 Organization and cell differentiation in lateral roots of *Arabidopsis thaliana* . Development 124, 33–44. 9006065 10.1242/dev.124.1.33

[CIT0038] Matsubayashi Y 2014 Posttranslationally modified small-peptide signals in plants. Annual Review of Plant Biology 65, 385–413. 10.1146/annurev-arplant-050312-120122 24779997

[CIT0039] Matsuzaki Y Ogawa-Ohnishi M Mori A Matsubayashi Y 2010 Secreted peptide signals required for maintenance of root stem cell niche in *Arabidopsis* . Science 329, 1065–1067. 20798316 10.1126/science.1191132

[CIT0040] Maule AJ Gaudioso-Pedraza R Benitez-Alfonso Y 2013 Callose deposition and symplastic connectivity are regulated prior to lateral root emergence. Communicative & Integrative Biology 6, e26531. 24563707 10.4161/cib.26531 PMC3917962

[CIT0041] Meng L Buchanan BB Feldman LJ Luan S 2012 CLE-like (CLEL) peptides control the pattern of root growth and lateral root development in *Arabidopsis* . Proceedings of the National Academy of Sciences of the United States of America 109, 1760–1765. 22307643 10.1073/pnas.1119864109 PMC3277184

[CIT0042] Moreno-Risueno MA Van Norman JM Moreno A Zhang J Ahnert SE Benfey PN 2010 Oscillating gene expression determines competence for periodic *Arabidopsis* root branching. Science 329, 1306–1311. 20829477 10.1126/science.1191937 PMC2976612

[CIT0043] Murphy E Smith S De Smet I 2012 Small signaling peptides in *Arabidopsis* development: how cells communicate over a short distance. The Plant Cell 24, 3198–3217. 22932676 10.1105/tpc.112.099010 PMC3462626

[CIT0044] Oelkers K Goffard N Weiller GF Gresshoff PM Mathesius U Frickey T 2008 Bioinformatic analysis of the CLE signaling peptide family. BMC Plant Biology 8, 1. 18171480 10.1186/1471-2229-8-1 PMC2254619

[CIT0045] Ohyama K Ogawa M Matsubayashi Y 2008 Identification of a biologically active, small, secreted peptide in Arabidopsis by *in silico* gene screening, followed by LC-MS-based structure analysis. The Plant Journal 55, 152–160. 18315543 10.1111/j.1365-313X.2008.03464.x

[CIT0046] Okamoto M Higuchi-Takeuchi M Shimizu M Shinozaki K Hanada K 2014 Substantial expression of novel small open reading frames in *Oryza sativa* . Plant Signaling & Behavior 9, e27848. 24526015 10.4161/psb.27848 PMC4091330

[CIT0047] Ouyang S Zhu W Hamilton J 2007 The TIGR Rice Genome Annotation Resource: improvements and new features. Nucleic Acids Research 35, D883–D887. 17145706 10.1093/nar/gkl976 PMC1751532

[CIT0048] Parizot B De Rybel B Beeckman T 2010 VisuaLRTC: a new view on lateral root initiation by combining specific transcriptome datasets. Plant Physiology 153, 34–40. 20219832 10.1104/pp.109.148676 PMC2862419

[CIT0049] Parizot B Roberts I Raes J Beeckman T De Smet I 2012 *In silico* analyses of pericycle cell populations reinforce their relation with associated vasculature in *Arabidopsis* . Philosophical Transactions of the Royal Society B: Biological Sciences 367, 1479–1488. 10.1098/rstb.2011.0227 PMC3321678 22527390

[CIT0050] Pearson WR 2000 Flexible sequence similarity searching with the FASTA3 program package. Methods in Molecular Biology 132, 185–219. 10547837 10.1385/1-59259-192-2:185

[CIT0051] Roberts I Smith S De Rybel B Van Den Broeke J Smet W De Cokere S Mispelaere M De Smet I Beeckman T 2013 The CEP family in land plants: evolutionary analyses, expression studies, and role in *Arabidopsis* shoot development. Journal of Experimental Botany 64, 5371–5381. 24179095 10.1093/jxb/ert331

[CIT0052] Roxrud I Lid SE Fletcher JC Schmidt ED Opsahl-Sorteberg HG 2007 GASA4, one of the 14-member *Arabidopsis* GASA family of small polypeptides, regulates flowering and seed development. Plant & Cell Physiology 48, 471–483. 17284469 10.1093/pcp/pcm016

[CIT0053] Saeed AI Sharov V White J 2003 TM4: a free, open-source system for microarray data management and analysis. BioTechniques 34, 374–378. 12613259 10.2144/03342mt01

[CIT0054] Schnable PS Ware D Fulton RS 2009 The B73 maize genome: complexity, diversity, and dynamics. Science 326, 1112–1115. 19965430 10.1126/science.1178534

[CIT0055] Schuster-Böckler B Bateman A 2005 Visualizing profile-profile alignment: pairwise HMM logos. Bioinformatics 21, 2912–2913. 15827079 10.1093/bioinformatics/bti434

[CIT0056] Seitz O 2000 Glycopeptide synthesis and the effects of glycosylation on protein structure and activity. ChemBioChem 1, 214–246. 11828414 10.1002/1439-7633(20001117)1:4<214::AID-CBIC214>3.0.CO;2-B

[CIT0057] Shinohara H Matsubayashi Y 2013 Chemical synthesis of Arabidopsis CLV3 glycopeptide reveals the impact of hydroxyproline arabinosylation on peptide conformation and activity. Plant & Cell Physiology 54, 369–374. 23256149 10.1093/pcp/pcs174 PMC3589827

[CIT0058] Silverstein KAT Moskal WA Jr Wu HC Underwood BA Graham MA Town CD VandenBosch KA 2007 Small cysteine-rich peptides resembling antimicrobial peptides have been under-predicted in plants. The Plant Journal 51, 262–280. 17565583 10.1111/j.1365-313X.2007.03136.x

[CIT0059] Smyth GK 2004 Linear models and empirical Bayes methods for assessing differential expression in microarray experiments. Statistical Applications in Genetics and Molecular Biology 3, Article 3. 10.2202/1544-6115.1027 16646809

[CIT0060] Somssich M Simon R 2012 Peptides regulating apical meristem development. In: Irving HR Gehring C, eds. Plant signaling peptides (Signaling and Communication in Plants, Vol. 16). Heidelberg: Springer, 25–39.

[CIT0061] Srivastava R Liu J-X Guo H Yin Y Howell SH 2009 Regulation and processing of a plant peptide hormone, AtRALF23, in Arabidopsis. The Plant Journal 59, 930–939. 19473327 10.1111/j.1365-313X.2009.03926.x

[CIT0062] Stahl Y Wink RH Ingram GC Simon R 2009 A signaling module controlling the stem cell niche in *Arabidopsis* root meristems. Current Biology 19, 909–914. 19398337 10.1016/j.cub.2009.03.060

[CIT0063] Tanaka T Antonio BA Kikuchi S 2008 The Rice Annotation Project Database (RAP-DB): 2008 update. Nucleic Acids Research 36, D1028–D1033. 18089549 10.1093/nar/gkm978 PMC2238920

[CIT0064] Tuskan GA DiFazio SP Jansson S 2006 The genome of black cottonwood, *Populus trichocarpa* (Torr. & Gray). Science 313, 1596–1604. 16973872 10.1126/science.1128691

[CIT0065] Vanneste S De Rybel B Beemster GTS 2005 Cell cycle progression in the pericycle is not sufficient for SOLITARY ROOT/IAA14-mediated lateral root initiation in *Arabidopsis thaliana* . The Plant Cell 17, 3035–3050. 16243906 10.1105/tpc.105.035493 PMC1276028

[CIT0066] Vie AK Najafi J Liu B Winge P 2015 The *IDA/IDA-LIKE* and *PIP/PIP-LIKE* gene families in *Arabidopsis:* phylogenetic relationship, expression patterns, and transcriptional effect of the PIPL3 peptide. Journal of Experimental Botany 66, 5351–5365. 10.1093/jxb/erv285 PMC4526919 26062745

[CIT0067] Whitford R Fernandez A Tejos R 2012 GOLVEN secretory peptides regulate auxin carrier turnover during plant gravitropic responses. Developmental Cell 22, 678–685. 22421050 10.1016/j.devcel.2012.02.002

[CIT0068] Zhang H Zhou H Berke L Heck AJR Mohammed S Scheres B Menke FLH 2013 Quantitative phosphoproteomics after auxin-stimulated lateral root induction identifies an SNX1 protein phosphorylation site required for growth. Molecular and Cellular Proteomics 12, 11581169. 10.1074/mcp.M112.021220 PMC3650328 23328941

