# Supplementary material for: Expanding the repertoire of secretory peptides controlling root development with comparative genome analysis and functional assays
Source: J Exp Bot. 2015 Jul 20;66(17):5257–69. doi: 10.1093/jxb/erv346 (PMC4526923; doi:10.1093/jxb/erv346)
Supplement: Supplementary Data [file supp_66_17_5257__index.html]

Expanding the repertoire of secretory peptides controlling root development with comparative genome analysis and functional assays — Supplementary Data 

# Expanding the repertoire of secretory peptides controlling root development with comparative genome analysis and functional assays

## Supplementary Data

Data files

- Supplementary Data - Supplementary Data
- Supplementary Data - Supplementary Data
